# New Arteriovenous Skin Rash in a Patient with ESKD on Dialysis

**DOI:** 10.34067/KID.0000000000000069

**Published:** 2023-03-30

**Authors:** Sonali Batta, Allie Preston, Lindsay Bicknell

**Affiliations:** 1 Texas A&M College of Medicine, Temple, Texas; 2 Baylor Scott & White Medical Center, Department of Dermatology, Temple, Texas

**Keywords:** dialysis, allergic contact dermatitis, chlorhexidine, dialysis

## Abstract

**Figure:**
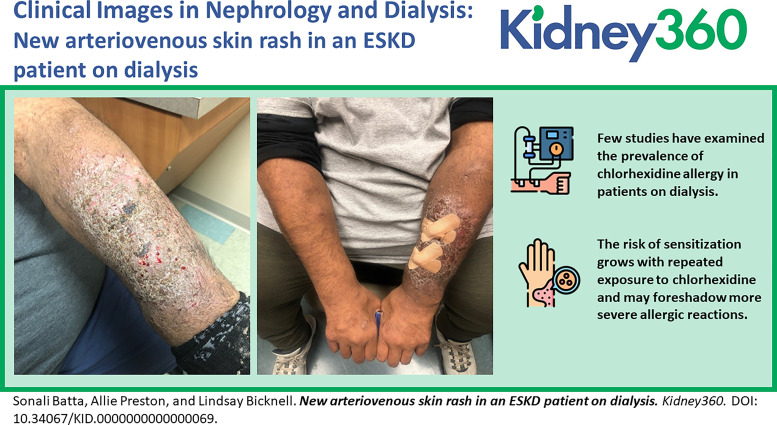

## Case Description

**Figure 1:**
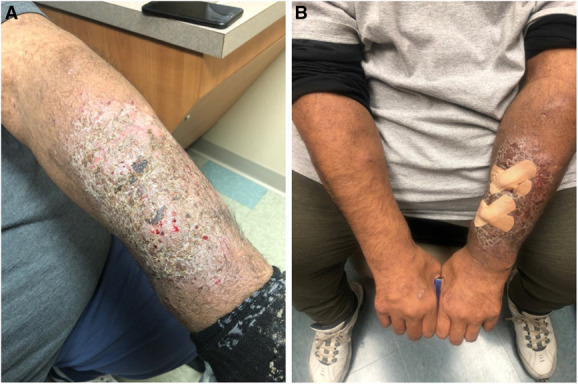
**Physical Exam**. (A) Well-circumscribed, lichenified, scaly plaque with underlying erythema overlying arteriovenous fistula site. (B) Comparison of findings on left versus right dorsal forearm.

A 53-year-old man with dialysis-dependent end-stage kidney disease, diabetes mellitus type II, and hypertension presented for evaluation of a pruritic rash located on his left dorsal forearm. The rash began after he transferred dialysis facilities approximately 4 months before presentation. According to the patient, his prior dialysis facility used povidone iodine to cleanse the fistula site prior to access while the current facility uses chlorhexidine. On physical examination, a well-circumscribed, lichenified, scaly plaque with underlying erythema was noted on the left dorsal forearm overlying his arteriovenous (AV) fistula site (Figure 1A). He denied rash or pruritus elsewhere (Figure 1B). Known drug allergies included ibuprofen. Given the well-circumscribed nature of the patient's eruption and temporal association with the change in his dialysis facility and antiseptic used, a diagnosis of allergic contact dermatitis secondary to chlorhexidine was established. Three weeks later, he reported significant improvement in both symptoms and appearance of the left arm after use of topical clobetasol and avoidance of chlorhexidine-containing products.

## Discussion

Chlorhexidine exposure has been linked to various hypersensitivity reactions.^1^ Allergic contact dermatitis is more common with chlorhexidine than with other antiseptics.^2^ Although several studies have reported chlorhexidine sensitization in health care workers, few have examined the prevalence in other groups frequently exposed to chlorhexidine, such as dialysis patients.^3^

Allergic contact dermatitis is a delayed T-cell–mediated hypersensitivity reaction that occurs in those with repeated allergen exposure.^4^ Acute allergic contact dermatitis presents as a localized erythematous, eczematous, or vesicular dermatitis in the area of exposure, whereas chronic allergic contact dermatitis manifests with lichenification, fissuring, and scaling. Reactions to chlorhexidine are enhanced when applied to damaged skin barriers, mucosal membranes, or with direct vascular exposure.^2^ Chlorhexidine intolerance is, therefore, more common among patients with AV grafts and AV fistulae than among those with central lines.^5^ In addition, subclinical thinning of the stratum corneum due to friction may increase systemic chlorhexidine absorption.^4^ This is particularly relevant for dialysis patients, whose skin barrier integrity is often compromised because of recurrent mechanical trauma and decreased epidermal water content.^2^ Hemodialysis patients are relatively anergic, which may reduce the symptoms of contact sensitization and result in isolated pruritus without other eczematous changes.^2^

Although initial irritation is often deemed insignificant, it may foreshadow more severe reactions on continued exposure because the risk of sensitization grows with repeated exposure and higher concentrations.^4^ In fact, perioperative anaphylaxis due to chlorhexidine has been documented in a patient with unknown chlorhexidine sensitization that occurred years before the anaphylactic event.^2^ Given the large number of dialysis patients who interact frequently with health care environments, there is a substantial risk for subsequent and sustained chlorhexidine exposure in this population. Therefore, facilities should retain a record of chlorhexidine-containing products. All practitioners should be mindful of potential sources of chlorhexidine and its allergic potential when caring for dialysis patients.

## Teaching Points

- Few studies have examined the prevalence of chlorhexidine allergy in patients on dialysis.
- The risk of sensitization grows with repeated exposure to chlorhexidine and may foreshadow more severe allergic reactions.

## References

[1] Chiewchalermsri C Sompornrattanaphan M Wongsa C Thongngarm T. Chlorhexidine allergy: current challenges and future prospects. J Asthma Allergy. 2020;13:127–133. doi:10.2147/JAA.S207980 32210588 PMC7069565

[2] Tan JN Da Y Haroon S Lau T. Chlorhexidine—a commonly used but often neglected culprit of dialysis associated anaphylactic reactions (case report). BMC Nephrol. 2022;23(1):18. doi:10.1186/s12882-021-02646-x 34991509 PMC8734226

[3] Gaudy-Marqueste C Jouhet C Castelain M Contact allergies in haemodialysis patients: a prospective study of 75 patients. Allergy. 2009;64(2):222–228. doi:10.1111/j.1398-9995.2008.01833.x 19178401

[4] Opstrup MS Jemec GBE Garvey LH. Chlorhexidine allergy: on the rise and often overlooked. Curr Allergy Asthma Rep. 2019;19(5):23. doi:10.1007/s11882-019-0858-2 30874959

[5] Kallen AJ Patel PR Hess S. Intolerance of chlorhexidine as a skin antiseptic in patients undergoing hemodialysis. Infect Control Hosp Epidemiol. 2011;32(11):1144–1146. doi:10.1086/662591 22011549

